# Molecular Advances in Male Infertility and Fertility: Importance of Redox Regulation and Oxidative Stress

**DOI:** 10.3390/ijms27093819

**Published:** 2026-04-25

**Authors:** Robert J. Aitken, Monica H. Vazquez-Levin, João S. Hallak, Thiago A. Teixeira, Jorge Hallak

**Affiliations:** 1 Centre for Reproductive Science, School of Science, University of Newcastle, Callaghan, NSW 2308, Australia; 2 Hunter Medical Research Institute, New Lambton Heights, Newcastle, NSW 2305, Australia; 3 Institute of Biology and Experimental Medicine (IBYME), Consejo Nacional de Investigaciones Científicas y Técnicas, Buenos Aires C1428ADN, Argentina; mhvazl@gmail.com; 4 Centro Universitário em Saúde, Faculdade de Medicina do ABC, Santo André 09060-060, SP, Brazil; joao.hallak@aluno.fmabc.net; 5 Instituto ANDROSCIENCE, de Ciência, Educação e Projetos Avançados em Saúde Masculina, São Paulo 04000-000, SP, Brazil; thafonsoteixeira@gmail.com (T.A.T.); hallakj@androscience.com.br (J.H.); 6 Disciplina de Urologia, Departamento de Cirurgia, Faculdade de Medicina, Universidade Federal do Amapá–UNIFAP, Macapá 66600-000, AP, Brazil; 7 Grupo de Estudos em Saúde Masculina, Instituto de Estudos Avançados, Universidade de São Paulo–IEA-USP, São Paulo 01230-000, SP, Brazil; 8 ANDROSCIENCE, Centro de Ciência e Inovação em Andrologia, Laboratório Clínico e de Pesquisa de Alta Complexidade, São Paulo 01310-000, SP, Brazil; 9 Departamento de Patologia, Unidade de Toxicologia Reprodutiva, Faculdade de Medicina da Universidade de São Paulo–FMUSP, São Paulo 01246-000, SP, Brazil; 10 Disciplina de Urologia, Departamento de Cirurgia, Hospital das Clínicas da Faculdade de Medicina da Universidade de São Paulo–FMUSP, São Paulo 01246-000, SP, Brazil

**Keywords:** male infertility, spermatozoa, oxidative stress, mitochondria, NADPH oxidase, L-amino acid oxidase, lipid peroxidation, DNA damage, antioxidants

## Abstract

Oxidative stress is one of the few defined causes of male infertility affecting at least one third of patients attending infertility clinics. Human spermatozoa are vulnerable to this form of attack because their stripped-down architecture means that they possess limited antioxidant protection and little capacity for biochemical repair. They also compound their vulnerability by being active generators of reactive oxygen species (ROS) and possessing multiple substrates for oxidative damage. The major sources of ROS in these cells are their mitochondria, an L-amino acid oxidase (IL4I1) and a calcium-dependent NADPH oxidase (NOX5). Spermatozoa tolerate the risks associated with ROS generation because their biology is heavily dependent on redox regulation. ROS are important mediators of sperm capacitation, stimulating the generation of cAMP and prostaglandins, inhibiting protein phosphatases and encouraging removal of cholesterol from the plasma membrane. Furthermore, during fertilization, the ability of ROS to activate metalloproteinases facilitates penetration of the zona pellucida and sperm–oocyte fusion. While ROS are physiologically important for sperm function, the over-production of these metabolites can impair sperm function. Antioxidants have therefore assumed some importance as a possible therapy for the infertile male. However, before this potential can be realized, we need to optimize the composition and dose of reagents used in such formulations and develop improved methods of diagnosing oxidative stress within the patient population.

## 1. Introduction

Male infertility is a relatively common condition affecting more than 10% of men worldwide and potentially making a significant contribution to the global decline in fertility rates [1,2,3,4]. However, despite the clinical significance of this condition in defining the reproductive health of our species, it should also be acknowledged that neither the true prevalence nor the detailed etiology of male infertility is fully understood. The traditional laboratory approach to diagnosing this condition involves using descriptive criteria to define key elements of the semen profile, with a focus on sperm motility, morphology, and count. The World Health Organisation (WHO) [4] has been instrumental in standardizing these seminal diagnostic criteria and in setting thresholds of normality based on detailed surveys of men with proven fertility. Notwithstanding the excellent contribution the WHO has made to the standardization of human semen analysis, the detailed description of a symptom should not be confused with the diagnosis of a disease. The semen profile is largely silent on causation and, as a result, the true contribution that male factors make to human infertility is cloaked in uncertainty. Given this lack of diagnostic awareness, men are often viewed merely as ‘sperm providers’ rather than patients with a potentially treatable clinical condition. In an Assisted Reproductive Technology (ART) context, this has led to a situation where ICSI (Intra-Cytoplasmic Sperm Injection) is being used as a universal therapeutic hammer to hit every kind of infertility nail, with little thought for the underlying etiology or the possibility of developing treatments that address the causes rather than the consequences of the patient’s condition. In all cases, the ultimate clinical aim is to restore normal fertility while avoiding potential medical complications in the offspring, and possibly future generations, as a result of highly invasive ART procedures [5,6].

Over the past half-century, the only genuine insights into the causation of male infertility have been genetics and oxidative stress [7]. In the case of genetics, we know that Klinefelter syndrome and microdeletions on the long arm of the Y chromosome are the major contributors to male infertility. However, with the passage of time, an increasing number of recessive (often biallelic) mutations are being identified that cause conditions such as globozoospermia, primary ciliary dyskinesia, congenital absence of the vas deferens, multiple morphological abnormalities of the sperm flagellum, macrozoospermia, and spermatogenic arrest [8,9]. These conditions remain mercifully rare despite the best efforts of the ART community to ensure that such genetic defects are retained within the population through the indiscriminate use of ICSI [6]. The problem with genetic causes of male infertility is that ICSI is often our only option—we have no choice. However, this is not the case with oxidative stress.

## 2. Oxidative Stress and Male Infertility

**Figure 1:**
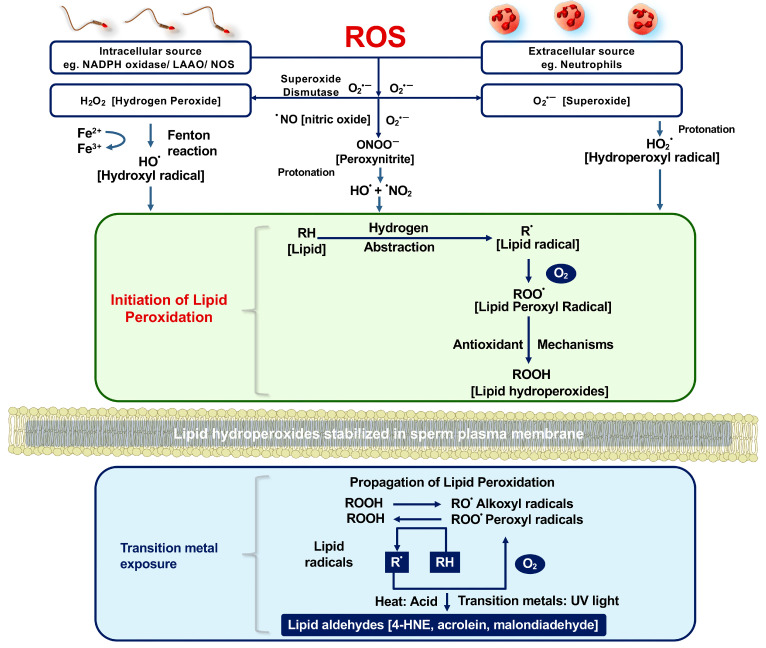
Spermatozoa and infiltrating leukocytes generate ROS primarily via NADPH oxidases supplemented, in the case of spermatozoa, by electron leakage from the mitochondria and activation of L-amino acid oxidase (LAAO) and nitric oxide synthase (NOS) activities. The major products are superoxide (O_2_^•−^), which rapidly dismutates to hydrogen peroxide (H_2_O_2_), and nitric oxide (^•^NO), which reacts with O_2_^•−^ to form peroxynitrite (ONOO^−^). Through a combination of Fenton chemistry and protonation reactions, these ROS secondarily generate highly reactive hydroxyl (HO^•^) and hydroperoxyl (HO_2_^•^) radicals. These radical species abstract hydrogen atoms from adjacent lipids, forming lipid radicals (R^•^), which react with oxygen to generate the corresponding lipid peroxyl radical (ROO^•^). This peroxyl radical then abstracts another hydrogen atom to stabilize as the lipid hydroperoxide (ROOH) and, in the process, creates another radical (R^•^), thereby propagating the lipid peroxidation cascade. In the presence of transition metals (e.g., Fe^2+^, Cu^+^), lipid hydroperoxides in the plasma membrane can be induced to form secondary electrophilic aldehydes (e.g., 4-hydroxynonenal) that impair membrane integrity, disrupt protein function, and fragment DNA. Endogenous antioxidants (enzymatic and nonenzymatic) are the primary defense against this form of damage.

Oxidative stress has been implicated in the etiology of male infertility since the pioneering studies of John McLeod in the 1940s describing the beneficial effects of catalase on sperm motility [10]. Subsequent studies have confirmed the importance of oxidative stress as a widespread cause of male infertility [7,11,12,13], stemming in part from the unique susceptibility of human spermatozoa to oxidative assault. Thus, spermatozoa are well endowed with targets vulnerable to ROS attack, including proteins, nucleic acids, carbohydrates, and lipids. The latter are particularly significant because ~50% of the fatty acids in a human spermatozoon is docosahexaenoic acid with 6 double bonds per molecule [11]. These double bonds are sensitive to free-radical attack at the bis-allylic carbon between them, where hydrogen dissociation energies are lowest. As a result, hydrogen abstraction is facilitated, triggering a lipid peroxidation cascade that severely compromises sperm function (Figure 1) [11,14].

Spermatozoa are also vulnerable to oxidative stress because, just before their release from the germinal epithelium, they shed most of their cytoplasm, retaining just a small amount in the sperm midpiece. This severely limits the availability of intracellular antioxidants to protect these cells from oxidative attack. As a result, human spermatozoa are highly dependent on the antioxidants provided in the extracellular space [11], which is why human seminal plasma is one of the richest antioxidant fluids known to man [15]. It is also why male infertility is commonly associated with a decline in seminal antioxidant protection [16].

Despite the architectural and biochemical vulnerabilities of human spermatozoa, these cells are also highly proficient producers of ROS, which are used to drive their complex physiology. This raises two obvious questions: (1) how are the ROS generated, and (2) if these cells are so vulnerable to oxidative stress, why do they do it?

## 3. Mechanisms of ROS Generation

### 3.1. Sperm Mitochondria

One of the major sources of ROS in mammalian spermatozoa is their mitochondria. In more than 90% of mammalian species, when females exhibit behavioral estrus, they are likely to be mated by multiple males, leading to intense competition within the female tract to be the first sperm to fertilize the egg(s). Under these circumstances, evolutionary selection pressure creates ejaculates characterized by large numbers of rapidly moving spermatozoa and a sperm physiology that depends on ATP generated by oxidative phosphorylation. Human spermatozoa are different. Neither behavioral estrus nor multiple matings are characteristic of our species, which generally favors a ‘serial monogamy’ reproductive strategy. As a result, there is no mechanism to synchronize mating with ovulation and little prospect of competition from the spermatozoa of other males. Following mating, human spermatozoa may therefore have to spend several days alone, patiently waiting in the Fallopian tubes for an egg to arrive. Human spermatozoa are therefore built for marathons rather than sprints and consequently divert much of their metabolism away from oxidative phosphorylation and towards glycolysis. Because there has been no selection pressure on human sperm mitochondria, these organelles are not as highly evolved as in other species, where sperm competition predominates [17]. Human sperm mitochondria are therefore inherently leaky and liable to shed electrons from their electron transport chain (ETC) that are then swept up by nature’s universal electron acceptor, oxygen, to generate superoxide anion (O_2_^−•^). Indeed, it has been found that around 70% of O_2_^−•^ generation by human spermatozoa emanates from their mitochondria [18] and that most damage is generated following ROS generation at Complex I [18,19].

### 3.2. L-Amino Acid Oxidase

**Figure 2:**
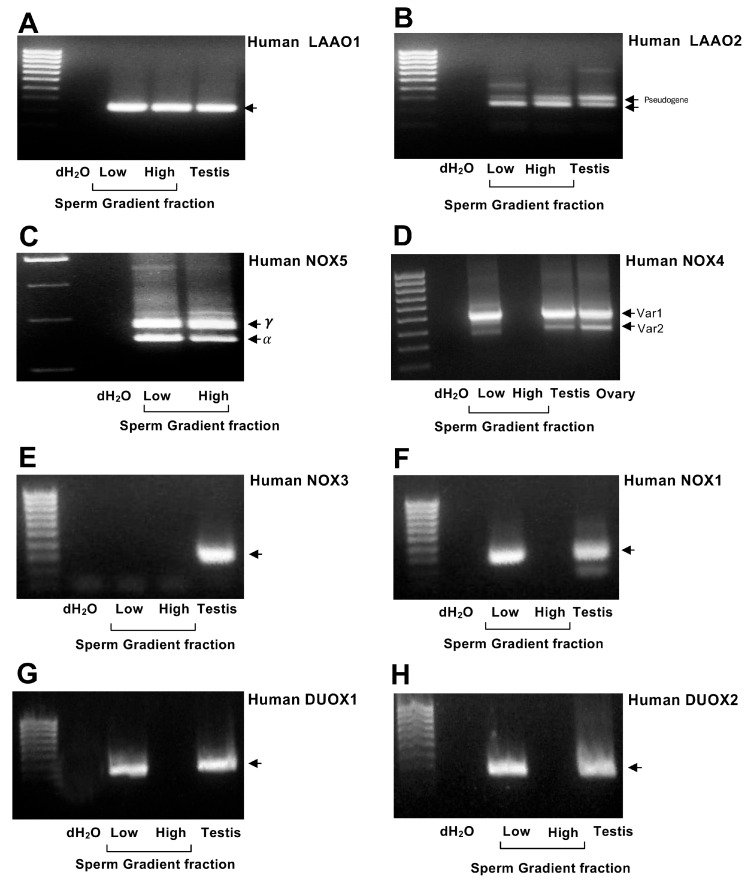
Nested PCR revealing the presence of mRNA for several ROS-generating enzymes in human spermatozoa and testes. (**A**) Transcripts for LAAO1 in human testes and spermatozoa. (**B**) Transcripts for LAAO2 in human testes and spermatozoa. (**C**) Transcripts for the α and γ isoforms of NOX5 in both high- and low-density sperm populations. (**D**) NOX4 transcripts in testes, ovaries, and low-density sperm suspensions but not in high-density purified spermatozoa populations. (**E**) NOX3 transcript in human testes but not spermatozoa. (**F**) mRNA for NOX1 in human testes and low-density sperm populations, but not in spermatozoa recovered from the high-density region of discontinuous density gradients. (**G**) Transcripts for DUOX1 in human testes and low-density sperm populations. (**H**) Transcripts for DUOX2 in human testes and low-density sperm populations. Panels (**A**,**B**) from reference [24] with kind permission from Bioscientifica. Panels (**C**–**H**) from reference [33] with kind permission from Springer-Nature.

The very first cell type shown to generate ROS at the cellular level was the spermatozoon. In a paper published 80 years ago in Nature [20], Tosic and Walton demonstrated that the addition of an egg yolk-based cryopreservation medium to bovine spermatozoa impaired motility via mechanisms that could be reversed by catalase. Four years later, the same authors showed that hydrogen peroxide generation under these conditions involved an L-amino acid oxidase (LAAO) that used aromatic amino acids as substrates (L-phenylalanine, L-tryptophan, L-tyrosine) [20]. L-amino acid oxidases have now been detected in the spermatozoa of all species that have been examined to date, including bull, ram, boar, stallion, mouse, and man [20,21,22,23,24,25]. In human spermatozoa, mRNAs for 2 isoforms of LAAO have been detected in both the testes and purified suspensions of human spermatozoa (Figure 2A,B), which generate ROS according to the following formula:

LAAO C_6_H_5_CH_2_CH(NH_2_)COOH + O_2_ + H_2_O ⟶ C_6_H_5_CH_2_COCOOH + NH_3_ + H_2_O_2_ Phenylalanine           Phenylpyruvic acid Ammonia

Evidence has been secured for both human [24] and mouse [22] spermatozoa, suggesting that this oxidase system is positively involved in generating the redox drive to capacitation (see below). In the microcosm of cryopreservation, when sperm suspensions are exposed to cryopreservation media rich in aromatic amino acids from egg yolk, it is the H_2_O_2_ generated by non-viable cells that attacks the functionality of live spermatozoa in the immediate vicinity. Treatments that destroyed or inhibited oxidase activity (e.g., *N*-ethylmaleimide, a thiol alkylating agent, or exposure to heat, acid, or alkali) were found to eliminate or inhibit the toxicity associated with egg-yolk exposure [21]. This knowledge ultimately led to the formulation of improved cryopreservation media that omitted the egg yolk component but incorporated antioxidants to reduce the oxidative stress associated with this procedure [26,27].

So, decades before the biomedical importance of redox chemistry was highlighted by the discovery that leukocytes could actively generate ROS during phagocytosis, gamete biologists were well aware that spermatozoa were active in this respect and that excessive production of such metabolites could be damaging to the physiological integrity of these cells.

### 3.3. NADPH Oxidase Activity

One of the most interesting and controversial sources of ROS in human spermatozoa involves a cohort of enzymes, including NADPH oxidases (NOXs) and Dual oxidases (DUOXs), that generate ROS using NADPH as the preferred electron donor [28] according to the formula:

NOX NADPH + 2O_2_    ⟶  NADP^+^ + H^+^ + O_2_^−•^ DUOX

In keeping with this general concept, exogenous NAD(P)H was found to stimulate ROS generation in purified populations of human spermatozoa through flavoproteins that could be inhibited by diphenylene iodonium (DPI) or quinacrine [29]. Subsequently, an NADPH oxidase was confirmed in these cells as NOX5, a flavoprotein enzyme with a unique N-terminal extension containing three EF hand motifs, conferring this oxidase with calcium sensitivity [30,31]. NOX5 is present in human spermatozoa, at both mRNA and protein levels, and may make a contribution to cellular ROS generation during sperm capacitation [32,33], although this role has not yet been definitively proven. Nested PCR reveals the presence of transcripts for both the α- and the γ-isoforms of NOX5 in purified sperm suspensions (Figure 2C). The α-isoform is biochemically active in generating ROS, whereas the γ- isoform is catalytically inactive and may constitute a mechanism for ensuring that NADPH oxidase activity in the male germ line is maintained at a low level. Exactly how significant a contribution NOX5 makes to the redox balance in mammalian spermatozoa is difficult to gauge because the inhibitors that have been used to dissect the importance of this enzyme in the overall redox regulation of human spermatozoa (DPI and apocynin) lack the necessary specificity for firm conclusions to be drawn [33,34]. It is also uncertain whether NOX5 is the only contributor to NADPH-dependent ROS generation in human spermatozoa. Thus, while NOX5 has been confirmed in human, stallion, and canine spermatozoa, the mouse and rat genomes do not encode NOX5, and yet there is abundant evidence for the redox regulation of sperm function in these species [35,36].

NOX2 components have been reported by immunoblot in goat [37], rat [38], mouse [39], and guinea pig [40] but not human [31] spermatozoa. We have also detected mRNA transcripts for NOX4 (including both the full-length transcript (variant 1) and a truncated splice variant (variant 2), which may serve as a dominant negative regulator of NOX activity), as well as NOX3 and NOX1 in human testes. However, no such transcripts were detected in purified human sperm suspensions (Figure 2D–F). Similarly, DUOX1 and DUOX2 mRNAs are present in human testes, but not in purified sperm suspensions (Figure 2G,H) [33]. Based on this information, human spermatozoa appear to depend primarily on their mitochondria, LAAO, and NOX5 to meet their redox requirements.

### 3.4. Nitric Oxide Synthase

Another free radical source in human spermatozoa is nitric oxide synthase (NOS), which generates a reactive nitrogen species (RNS), nitric oxide (NO), from L-arginine using O_2_ and NADPH as co-substrates according to the formula:

2L-arginine + 3NADPH + 3H^+^ + 4O_2_ ⟶ 2citrulline + 2NO + 4H_2_O + 3NADP^+^

The NO generated by mammalian spermatozoa seems to be functionally important since incubation of spermatozoa with NG-nitro-L-arginine methyl ester (L-NAME), an NO synthesis inhibitor, has been shown to suppress capacitation, acrosomal exocytosis and sperm–oocyte fusion in a wide variety of species including human [41,42,43,44], mouse [45], hamster [46], boar [47], bull [48], and buffalo [49]. Interestingly, the activation of NO synthase in human spermatozoa can be promoted by the presence of citrate [50] and sphingosine 1-phosphate [51] and appears to involve association with two key proteins: sperm acrosome-associated 7 protein and retinoblastoma tumor-suppressor binding protein [52]. Despite this evidence for a positive role for NO in promoting sperm function, the overproduction of this RNS is known to have a suppressive effect [53,54]. Such negative impacts may involve the ability of NO to react very rapidly with O_2_^−•^ to generate peroxynitrite (ONOO^−^) an extremely powerful oxidizing agent (Figure 1). This molecule is known to be toxic to spermatozoa in high doses, while compounds that catalyze the decomposition of ONOO^−^ have been found to promote sperm survival [55]. Peroxynitrite is, therefore, yet another important component of the ROS repertoire that characterizes human spermatozoa. Given the potential toxicity of such ROS towards human spermatozoa, we might reasonably ask what physiological purpose is served by the generation of such highly reactive oxygen metabolites? What possible benefit could spermatozoa derive from ROS/RNS production that makes the risk inherent in such activity worthwhile?

## 4. Physiological Role of ROS in Sperm Biology

### 4.1. Chromatin Cross-Linking

Eutherian spermatozoa are characterized by a sperm head possessing extremely densely packaged chromatin, within which the DNA attains a near-crystalline state. Such effective packaging is achieved in the epididymis by the formation of multiple intra- and inter- molecular cross-bridges between cysteine residues involving positively charged nuclear proteins known as protamines [56]. The creation of these disulfide bridges also gives the sperm head a lance-like rigidity that allows spermatozoa reaching the vicinity of the egg to physically penetrate the zona pellucida [57], a thick acellular shell surrounding the ovulated ovum. The oxidation of protamine thiols in the epididymis is mediated by a peroxidase (glutathione peroxidase 4) embedded within the sperm chromatin, using hydrogen peroxide as the electron acceptor. The source of the hydrogen peroxide for this oxidation event is not known with certainty, but could involve the sperm mitochondria, the epididymal epithelium, or the resident leukocyte population [58,59,60].

### 4.2. Cholesterol Removal from the Plasma Membrane

At ejaculation, spermatozoa are vigorously motile but incapable of actually fertilizing the oocyte. At this point in their life history, they are said to exist in an ‘uncapacitated’ state. During their subsequent ascent of the female reproductive tract, spermatozoa become ‘capacitated’, whereby they acquire the functional attributes needed to achieve fertilization [61]. Capacitation is a complex process involving changes in the way spermatozoa move as well as alterations in the surface expression and location of receptor complexes that allow this highly differentiated gamete to distinguish the oocyte from any of the other 35 trillion cells in the human body. A key feature of capacitation is the increase in sperm membrane fluidity, which facilitates the exposure and lateral redistribution of egg receptor complexes to the anterior region of the sperm head, positioning them optimally for interaction with the oocyte surface [62,63,64]. In uncapacitated spermatozoa, the plasma membrane is a rigid, stable structure that prevents the sperm cell from undergoing the changes needed to interact successfully with the oocyte. This rigidity is maintained by cholesterol, which must be removed from the plasma membrane before the preparations for fertilization can begin [65]. This critical cholesterol-removal process is a redox-mediated event, driven by the ability of ROS to oxidize cholesterol and generate oxysterols. Owing to their increased hydrophilicity, these oxysterols migrate away from the hydrophobic core of the membrane toward the sperm surface, where they become accessible to and can be removed by sterol-binding proteins such as albumin [66]. Cholesterol efflux subsequently enhances membrane fluidity, enabling the exposure and redistribution of key receptors. This remodeling confers upon the plasma membrane the dynamic properties required for acrosomal exocytosis and, ultimately, for fusion with the oocyte.

### 4.3. Fatty Acid Oxidation

One of the key biochemical properties of ROS is their ability to induce lipid peroxidation. Generally, this is considered a destructive process that can destabilize membranes and lead to a loss of cellular integrity. However, this is not invariably the case; sometimes lipid peroxidation events are physiologically important, not least in the production of eicosanoids. These powerful biological mediators are generated by the oxidation of arachidonic acid under the influence of ROS and specific enzymes (cyclooxygenases and lipoxygenases), which yield prostaglandins, leukotrienes, and thromboxanes. Prostaglandins of the E-series (PGE_1_ and PGE_2_) are known to play an important role in driving sperm capacitation through the induction of calcium influx via the CatSper channel and the stimulation of cAMP generation [67,68,69,70,71,72]. It is also possible that PGE_2_ can propagate eicosanoid generation via the activation of phospholipase A_2_, which cleaves arachidonic acid from the *sn*-2 position of membrane phospholipids and makes it available for cyclooxygenase and lipoxygenase action [73]. The involvement of lipoxygenase in human sperm biology has been implicated by experiments using inhibitors to suppress leukotriene generation and demonstrating suppression of key biological events such as the acrosome reaction [73]. However, more direct evidence of leukotriene involvement in the regulation of human sperm function has not yet been secured. Human spermatozoa can also generate thromboxanes via the regulated oxidation of arachidonic acid [74], although, again, the specific role of these metabolites in sperm biology is largely unknown.

### 4.4. Protein Phosphorylation

Perhaps the most powerful impact of ROS on spermatozoa, as well as other cell types, is their ability to promote protein phosphorylation. One of the key pathways by which such stimulation is achieved is via the inactivation of protein phosphatases and, in the case of sperm function, the suppression of tyrosine phosphatases in particular. This action is achieved via the ability of powerful oxidants such as ONOO^−^ and H_2_O_2_ to oxidize catalytic cysteine residues at the active site of tyrosine phosphatases to generate sulfenic acid, which can then react with adjacent cysteines to form disulfide bonds, or with nearby amides to form sulfenyl-amide linkages [75]. As a result of this ROS-mediated suppression of tyrosine phosphatase activity, spermatozoa exhibit a global increase in tyrosine phosphorylation during capacitation [76,77]. This increase in phosphotyrosine expression is widely considered a hallmark of physiological sperm capacitation, even if, under some experimental conditions, it can be shown to be neither sufficient nor essential for spermatozoa to attain a capacitated state [78,79]. However, it is a good biomarker for a process that underpins several of the changes in sperm function needed to achieve fertilization. Thus, the induction of hyperactivated motility (a specific form of movement which is expressed as a result of sperm capacitation and serves to generate the propulsive forces needed to penetrate the egg’s zona pellucida), is directly induced by redox- mediated changes in tyrosine phosphorylation on A kinase anchoring proteins (AKAPs) in the sperm tail, as well as elevations in intracellular calcium and pH [80]. In some species, such as the mouse, tyrosine phosphorylation also contributes to remodeling the sperm surface to facilitate sperm–egg recognition [81], although the evidence for such a role in human fertilization is less compelling [82].

The impact of ROS on the tyrosine phosphorylation events occurring during sperm capacitation may not only involve the suppression of tyrosine phosphatase activity. In addition, ROS may also stimulate the generation of intracellular cAMP, the second messenger responsible for promoting tyrosine kinase activity. The ability of ROS to enhance cAMP levels has been reported in rat [83], bull [84], and human [85,86] spermatozoa but not in mouse [87].

Mammalian spermatozoa possess a type 10 soluble adenylyl cyclase (sAC) that is known to be activated by bicarbonate [88]; however, in the spermatozoa of certain species, ROS are just as effective in the promotion of cAMP generation as this anion [84]. Although the mechanisms underpinning redox-regulated cAMP generation are not fully understood, sAC contains specific cysteine residues that are susceptible to oxidation, forming disulfide bonds or sulfenic acids. These modifications may induce a structural shift in the catalytic domain, increasing the enzyme’s affinity for its substrate, ATP, or its essential cofactors, Mg^2+^ or Mn^2+^. It is also possible that ROS can enhance carbonic anhydrase activity, thereby increasing intracellular bicarbonate ion concentration and indirectly stimulating sAC [89].

ROS also inhibit serine/threonine phosphatases via the same cysteine oxidation mechanism [90,91,92]. Human spermatozoa contain phosphatases, PP1, PP2B, and PP2A, the activity of which decreases during sperm capacitation, allowing serine/threonine phosphorylations to occur, largely in response to cAMP—mediated activation of protein kinase A (PKA). Targets for PKA-mediated phosphorylation during capacitation include the AKAPs (also mentioned above as major targets for tyrosine phosphorylation), various heat shock proteins, ion channels and transporters, such as CatSper and Slo3, cytoskeletal proteins including tubulin, metabolic enzymes, signal transduction mediators such as Src, and the 26S proteosome [93,94,95].

### 4.5. Protease Activation

ROS are also involved in stimulating protease activity, which plays a key role during capacitation and subsequent interaction with the oocyte. Most of these enzymes are located in the cell in an inactive state, either as pro-enzymes (zymogens such as proacrosin or plasminogen) or, in the case of metalloproteinases (MMPs), through their association with Tissue Inhibitors of Metalloproteinases (TIMPS) [96,97]. The low levels of ROS generated during sperm preparation for fertilization appear to make a significant contribution to the stimulation of protease activity. This may occur through ROS-mediated changes in phosphoprotein expression, such as activation of the 26S proteosome via PKA-mediated phosphorylation of the pS14-Rpn6 (serine 14-phosphorylated Rpn6) subunit [98]. Alternatively, the activation of pro-enzymes can occur through the oxidation of thiol groups to the corresponding sulfenic acid, leading to changes in protein configuration and allosteric exposure of the enzyme’s active site. This cysteine switch mechanism is responsible for activating metalloproteinases within the metzincin clan, several of which are involved in regulating sperm function, including MMP-2 and ADAM (A Disintegrin And Metalloproteinases) proteins such as ADAM20 and Fertilin β [99,100]. The latter contain disintegrin domains that act as ligands for integrins on the oocyte surface and form part of the mechanism that brings sperm and egg into close proximity at fertilization [101]. At higher levels of ROS exposure, such conformational changes can facilitate the proteolytic cleavage of the propeptide by other proteases and the consequent activation of enzyme activity. MMP activity can also be enhanced by ROS-mediated damage to regulatory TIMP proteins, which contain cysteine residues that are vulnerable to oxidative attack [100].

### 4.6. ROS and Fertilizing Capacity

Taken together, it is apparent that ROS are involved in many signal transduction pathways that drive spermatozoa into a state of capacitation, during which they acquire the capacity to recognize and fertilize the oocyte [101]. Once a capacitated state has been attained, ROS are also involved in the mediation of sperm-zona recognition [102,103], acrosomal exocytosis [104], and sperm–oocyte fusion [105]. Because of this physiological involvement, the entire tyrosine phosphorylation cascade, as well as the acrosome reaction and sperm–oocyte fusion, can be disrupted by antioxidants. Catalase is particularly effective in this context, suggesting a central role of hydrogen peroxide in orchestrating this process [84,87,106]. Such results are clinically important because manufacturers are currently adding antioxidants to in vitro fertilization media to reduce oxidative damage to gametes and embryos and to ensure the health and well-being of offspring [107]. Such strategies are rational in cases of male factor infertility where and when signs of oxidative stress are clearly evident [108]. However, in the absence of oxidative stress, excessive supplementation of culture media with antioxidants may be detrimental, as it can suppress the very biological processes we are trying to promote, by inducing reductive stress [109].

### 4.7. Apoptosis/Ferroptosis

A final physiological function mediated by oxidative stress in spermatozoa is cell death. These highly differentiated cells possess a truncated capacity to undergo apoptosis should sperm quality and survival be severely compromised. In this way, nature tries to ensure that only the highest quality gametes can survive the long journey from the cauda epididymis to the ampullary region of the Fallopian tubes and engage in fertilization. An odyssey that begins with a total of around 200 million ejaculated spermatozoa ends with only around 250 competing for fertilization of the oocyte [110]. In the end, every one of these 200 million suitors will meet an apoptotic fate, except for the single spermatozoon that achieves a measure of immortality by fertilizing the oocyte. Indeed, it has been argued that apoptosis is the default position for spermatozoa; like keratinocytes, these cells are born to die [111]. The interesting question, therefore, is not what induces apoptosis in human spermatozoa, but what prevents it. Like many somatic cells, the vitality of human spermatozoa depends on exposure to prosurvival factors that maintain the PI3K (Phosphoinositide 3-kinase)/AKT phosphorylation pathway in a highly active state [111]. As long as this signal transduction pathway is operating, effectors of apoptosis, such as BAD (Bcl-2-associated Agonist of Cell Death), are inactivated and sequestered in the cytoplasm by their 14-3-3 keeper proteins. As soon as the PI3K/AKT pathway ceases to function, BAD becomes dephosphorylated, is released from its 14-3-3 anchor, and moves to the mitochondria, where it antagonizes BCL2 and initiates the intrinsic apoptotic cascade. The pro-survival factors responsible for maintaining spermatozoa in a viable state have not been completely characterized, although prolactin [112], brain-derived neurotrophic factor [113], nerve growth factor [114], Fibroblast Growth Factor 2 [115], Glucagon-like peptide-1 (GLP-1) [116], insulin-like growth factors [117,118] and insulin itself [119,120] all appear to be involved. Indeed, insulin is so important for sperm survival that the male germ line synthesizes and packages its own supplies of insulin prior to ejaculation in order to support the survival of these cells during ascent of the female reproductive tract. More than this, the endometrium also manufactures and secretes insulin into the uterine lumen during the preovulatory phase of the cycle, thereby ensuring that spermatozoa are continuously supported by powerful prosurvival factors in the extracellular space [119].

The combined action of these prosurvival factors ensures the PI3K/AKT pathway remains fully active and sperm survival is assured during the challenges these cells will face on their quest to fertilize the oocyte, which, in our species, can be a journey of several days’ duration. However, spermatozoa do not last forever. When prosurvival factors like insulin are not available, or the signal transduction pathways utilized by such factors lose effectiveness, the PI3K/AKT pathway is no longer supported, and the cells shift into an early phase of apoptosis [111]. As a result of such changes, the plasma membrane Ca^2+/^Mg^2+^ ATPases that maintain intracellular calcium homeostasis are no longer maintained in a phosphorylated state, and extracellular calcium leaks into the cell [121]. The increased intracellular calcium triggers ROS generation by NOX5 [31] and the mitochondria [122], which further compromises ATPase function, dissipates mitochondrial membrane potential, decreases intracellular ATP and cAMP, and destabilizes the plasma membrane [122]. Via such mechanisms, the combination of rising intracellular calcium and enhanced ROS generation drives the intrinsic apoptotic process to completion [122,123].

**Figure 3:**
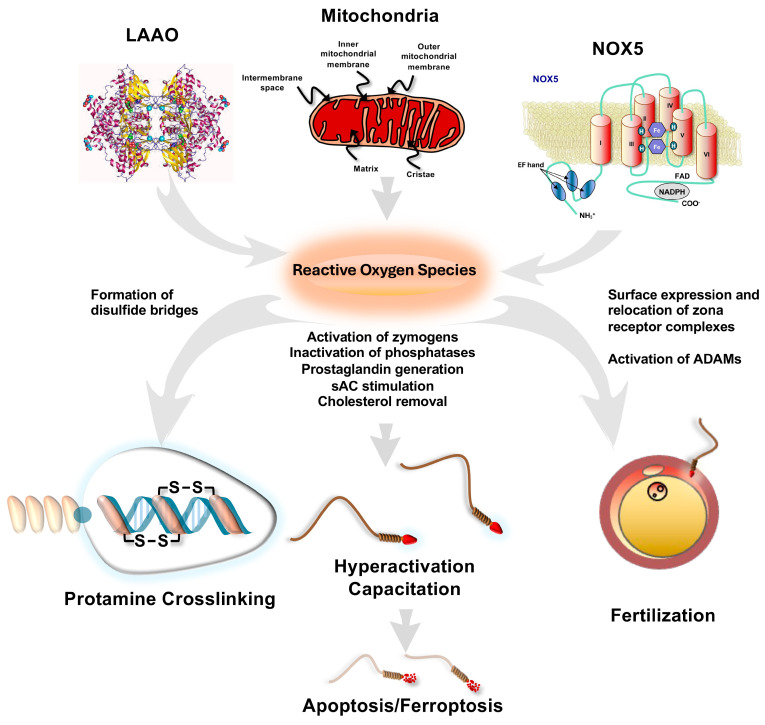
Sources and roles of ROS in human sperm biology. Major sources of ROS are LAAO, mitochondria, and NOX5. These oxygen metabolites then control various aspects of sperm development and function. In the epididymis, the chromatin in the sperm head becomes stabilized via the formation of inter- and intramolecular disulfide bridges. Post-ejaculation, ROS promote capacitation by activating zymogens, stimulating PGE production, promoting cAMP generation, and enhancing cholesterol removal to increase plasma membrane fluidity. This allows the receptor complexes responsible for sperm–egg recognition to be assembled, exteriorized, and moved to the anterior part of the cell. ROS also activate ADAM proteins, which mediate sperm–oocyte interaction. Those spermatozoa that do not achieve fertilization are destined to undergo a ROS-mediated apoptotic process prior to silent phagocytosis within the female tract.

Interestingly, controlled increases in intracellular calcium and ROS generation are also central to the induction of capacitation [124]. It has therefore been suggested that capacitation and senescence are at opposite ends of a continuum driven by oxidative stress [125]. Following ejaculation, the continuous production of low levels of ROS and a progressive increase in intracellular calcium levels lead to the attainment of a capacitated state [111]. However, if an egg does not arrive, the continued increase in ROS and intracellular calcium impairs the PI3K/AKT pathway via the mechanisms outlined above, and the cells enter a state of senescence that culminates in an apoptotic death characterized by enhanced mitochondrial ROS generation, lipid peroxidation, motility loss, caspase activation, and phosphatidylserine externalization [111] (Figure 3).

The regulated apoptotic demise that awaits virtually every sperm cell is biologically important because it results in markers on the sperm surface, such as phosphatidylserine, which inform the neutrophils and macrophages infiltrating the female reproductive tract following insemination that the phagocytic removal of senescent, moribund spermatozoa should be a silent process, unaccompanied by the generation of proinflammatory cytokines and ROS [126].

## 5. Disturbed Redox Balance and Male Infertility

Given the important physiological role of regulated ROS generation in creating a functional spermatozoon, it is now of interest to consider how this regulation is disturbed in cases of male infertility.

### 5.1. Abnormal Mitochondrial ROS Generation

At the cellular level, a common cause of oxidative stress is the increased generation of ROS by sperm mitochondria. A variety of factors are known to impede the smooth passage of electrons along the mitochondrial ETC and directly promote O_2_^−•^ generation. These factors include electromagnetic energy, in the form radio-frequency electromagnetic radiation (RFEMR) [127] or heat [128], as well as hydrophobic amphiphilic compounds that can insinuate themselves into the inner mitochondrial membrane and disturb electron flow, including free unesterified unsaturated fatty acids [129,130], certain polyphenolic food supplements [131], as well as environmental toxicants like bisphenol A [132] and phthalate esters [133]. Soluble products from bacteria such as *Escherichia coli* can also trigger mitochondrial ROS generation [134], as can any stressor driving the intrinsic apoptotic cascade, including prolonged storage both in vivo (e.g., as a result of low ejaculation frequency [135]), and in vitro (e.g., as a consequence of cryopreservation procedures [136]). In addition, enhanced mitochondrial ROS generation may be a developmental defect originating during spermatogenesis as a result of exposure to a range of factors such as obesity [137], bacterial infection [138], diabetes [139], or exposure to toxicants such as microplastics [140,141], arsenic [142] and endocrine disruptors including diethylstilbestrol and bisphenol A [143].

One of the reasons why mitochondrial ROS generation makes such a powerful contribution to oxidative stress in the male germ line is that it is a self-perpetuating process. As soon as oxidative stress is initiated, these cells (regardless of cause), the electrophilic aldehydes generated as a result of lipid peroxidation, including 4-hydroxynonenal (4-HNE) or acrolein, bind to cysteine-containing proteins in the mitochondrial ETC, such as succinic acid dehydrogenase [144]. The adduction of these proteins with aldehydes disturbs the transfer of electrons between the mitochondrial redox centers, leading to electron leakage and the generation of ROS, including O_2_^−•^ and H_2_O_2_. The latter then accelerates lipid peroxidation, generating yet more aldehydes that adduct proteins in the ETC and stimulate further mitochondrial ROS generation in a self-reinforcing cycle (Figure 1).

### 5.2. Abnormal NOX/NOS Activity

Aside from mitochondrial electron leakage, intrinsic ROS generation may also involve excessive activity on the part of the calcium-dependent NADPH oxidase, NOX5. Thus, asthenozoospermic patients with low sperm motility are characterized by overexpression of NOX5 [145]. Interestingly, the same patients also showed increased phospho-c-Abl expression, indicating that this was not a specific defect in NOX5 expression. A plausible explanation for these findings is that the elevation of NOX5 expression is the result of a developmental defect leading to the retention of excess residual cytoplasm in the midpiece of the spermatozoa.

Quantification of this morphological feature has revealed positive correlations between ROS generation and the increased presence of several cytoplasmic enzymes (superoxide dismutase, creatine kinase, and glucose-6-phosphate dehydrogenase (G6PD)), all of which were negatively correlated with sperm motility [146]. Most of these cytoplasmic enzymes are simply passengers, serving as passive markers of the cytoplasmic space. The black sheep in the family may be G6PD, which, by catalyzing the generation of NADPH via the hexose monophosphate shunt, provides NOX5 with the substrate that it needs to generate excessive ROS. This explanation helps us understand why spermatozoa go to such lengths to limit their cytoplasmic space; it imposes a physical limit on the amount of NADPH substrate available to NOX/NOS enzymes, permitting the generation of sufficient ROS/RNS to drive capacitation without inducing oxidative damage. In normal spermatozoa, any NADPH generated during the early stages of capacitation by the hexose monophosphate shunt would be readily scavenged by glutathione reductase (GR), which has a *K*_m_ for NADPH of approximately 10–20 µM. This enzyme works in tandem with glutathione peroxidase (GPx) to protect spermatozoa from lipid peroxidation as they engage the capacitation process [147,148,149,150]. The reduced glutathione (GSH) generated as a result of GR activity is also used by glutathione-S-transferase Pi to reduce oxidized cysteine residues in the peroxiredoxin (PRDX) family of antioxidants, to ensure that the redox drive to capacitation does not result in cellular damage [151,152]. In this context, PRDX6 is thought to be the major regulator of redox signaling and antioxidant protection in human and animal spermatozoa, by virtue of its intrinsic peroxidase and phospholipase A2 (PLA2) activities [153,154,155]. The PLA2 activity is thought to be particularly important for the protection of human spermatozoa since it not only releases the oxidized fatty acid for further processing by antioxidant enzymes, like GPx, but also (directly or indirectly) creates lysophosphatidic acid, which, through the mediation of G-protein coupled receptors, stimulates the PI3K/AKT pathway to maintain cell viability [152,155].

However, as capacitation proceeds, the progressive rise in intracellular calcium is associated with the activation of NOX5 and the enhanced generation of ROS. The latter ultimately overwhelms the GPx/PRDX protective systems to create a more oxidative environment capable of accelerating the spermatozoa towards full capacitation and a readiness to acrosome react in response to signals generated by the cumulus-oocyte mass [151]. In the presence of excess residual cytoplasm, the increased substrate (NADPH) available to calcium- activated NOX5 means this tipping point is reached earlier, leading to accelerated capacitation and early entry into senescence.

**Figure 4:**
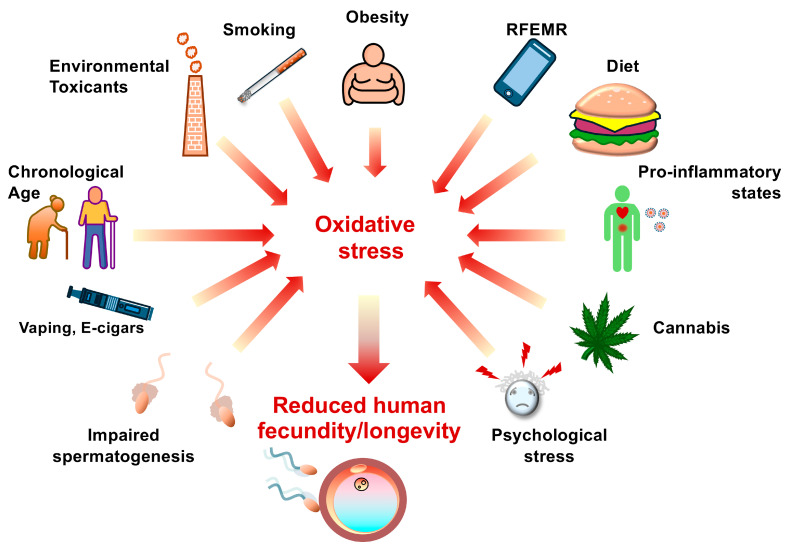
A wide range of factors encountered in modern industrialized society can enhance ROS generation and induce oxidative stress in the male germ line. These factors include disrupted spermatogenesis leading to excess cytoplasmic retention, vaping and E-cigars, aging, environmental toxicants such as bisphenol A, smoking, obesity, radio frequency electromagnetic radiation (RFEMR), nutrient-depleted diets, pro-inflammatory states such as varicocele and infection caused by SARS-CoV-2 and other viruses, recreational drugs including cannabis and alcohol, and psychological stress. The impact of such factors reduces human fertility and may also impact longevity.

In principle, the retention of excess residual cytoplasm can also be viewed as a Sertoli cell defect, since it is these cells that are responsible for removing the cytoplasm from differentiating spermatozoa during the final stages of spermiogenesis. So, any lifestyle, clinical, or environmental factor that affects Sertoli cell function could ultimately lead to excess cytoplasmic retention by spermatozoa and super-physiological ROS generation [156,157,158] (Figure 4).

### 5.3. Excess Leukocytic Infiltration

In addition to internal sources of ROS, such as electron leakage from the mitochondria or excessive enzyme activity, human spermatozoa are also vulnerable to extrinsic sources of free radicals. Most commonly, this involves the generation of ROS by activated neutrophils, which first come into contact with spermatozoa at the moment of ejaculation. Every human ejaculate is contaminated with phagocytic leukocytes (neutrophils and macrophages), largely originating from the secondary sexual glands and urethra. Their presence is to some extent physiological, facilitating the detection and elimination of moribund cells or invading organisms [159]. Although these leukocytes are activated and generate ROS, human spermatozoa are protected in vivo by the powerful antioxidants present in seminal plasma [160]. However, if the levels of leukocytic infiltration are pathological (>1 × 10^6^/mL) or even subclinical (>0.2 × 10^6^/mL), then these defenses may be overwhelmed, and parameters of semen quality, such as DNA integrity and motility, may be adversely affected [160,161,162,163]. In vitro, however, the story is different. If leukocytes are not carefully removed from the washed human sperm suspensions used for in vitro fertilization, the ROS they generate are free to attack the spermatozoa, inducing oxidative stress and limiting their fertilization capacity—even when leukocyte infiltration is low [164]. Removal of contaminating leukocytes from washed sperm suspensions using, for example, magnetic beads coated in antibodies against the common leukocyte antigen, reduces levels of oxidative stress and enhances fertilization rates [165].

### 5.4. Co-Morbidities and Pro-Inflammatory States

Oxidative stress in the male germ line can also be enhanced by a wide range of factors that encourage the development of a pro-inflammatory state including varicocele, obesity, hyperthermia, infection, poor diet, obstruction, toxicant exposure, psychological stress, smoking, aging, diabetes, as well as viral infections with multi-organ damage and systemic repercussions, like COVID-19 [166,167,168,169,170,171,172,173,174,175] (Figure 4).

In all these situations, ROS generation and lipid peroxidation are enhanced, while the overall antioxidant protection is diminished. Consequently, there is a strong relationship between the total antioxidant capacity measured in human semen and sperm function [176]. Moreover, the total antioxidant capacity in blood has been found to correlate with the antioxidant protection offered by seminal plasma and with elements of semen quality, including sperm count and motility [177]. These important observations suggest that the oxidative stress responsible for male infertility may reflect a more generalized redox imbalance that could affect other aspects of health, apart from fertility. Such relationships may explain the curious relationship that has been discovered between semen quality in young men and their ultimate longevity. Indeed, this may be why semen quality is considered a canary in the coal mine of male health; it is providing information that reflects the patient’s overall level of oxidative stress and hence their vulnerability to a variety of redox-regulated pathological conditions that might influence lifespan (cardiovascular disease, diabetes, neurodegeneration, cancer, respiratory disorders, renal failure, etc.), in addition to the suppression of sperm function and infertility [178,179].

## 6. Antioxidant Deficiency and Supplementation

Given the importance of antioxidant protection in controlling levels of oxidative stress, it is not surprising that the dietary intake of nutritious, antioxidant-rich food plays a key role in the ebb and flow of male infertility (Figure 4). Unhealthy, hyper-calorific diets, high in saturated and trans fats, are detrimental to semen quality, while diets rich in polyunsaturated fatty acids are beneficial [180]. Such considerations have fueled a massive growth industry in the design and administration of antioxidants for male infertility. There is a wide variety of formulations currently on the market containing various types of antioxidant compounds, in various doses, in the hope of correcting the oxidative stress responsible for around a third of all male infertility cases [181]. Unfortunately, very little thought has gone into the composition of these antioxidant formulations, and very few have been validated in vivo. To the authors’ knowledge, the sole exception is a commercial product that has been validated in two mouse models: the GPx5 knockout mouse and a scrotal heat-stress model [181]. The GPx5 knockout mouse exhibits elevated oxidative stress in the epididymis and the formation of 8-Oxo-2′-deoxyguanosine adducts in sperm DNA. Treatment with an antioxidant formulation for eight weeks completely protected the spermatozoa against oxidative DNA damage. Similarly, pre-treatment of mice with the antioxidant formulation significantly increased pregnancy rates in the heat stress model [181].

In view of such promising data, antioxidant trials have been conducted to confirm that antioxidant supplementation is a rational therapy for men experiencing infertility as a result of oxidative stress. Unfortunately, the clinical trials conducted to assess this potential have largely been an egregious waste of time, as patients have not been selected on the basis of oxidative stress. They have been selected because they are attending an infertility clinic, because they have poor sperm motility, because DNA fragmentation rates are high, etc., anything, in fact, other than some indication that the patients are actually suffering from a ROS-mediated pathology. How can we expect antioxidant therapy to be effective if the etiology of the patients’ infertility does not involve ROS-mediated cellular damage? Giving powerful antioxidants to patients who do not require them risks inducing reductive stress, which can be just as harmful as its oxidative counterpart [182]. So why do we do it?

One of the major reasons given for not using the measurement of oxidative stress as a selection criterion for such clinical trials is the absence of a clinically validated test for oxidative stress in males. A variety of approaches have been used to achieve this aim, although they all have shortcomings of one form or another.

## 7. Diagnosis of Oxidative Stress

### 7.1. Chemiluminescence

Chemiluminescent measurement of ROS generation is simple, inexpensive, and responsive, particularly when sensitized for H_2_O_2_ using horseradish peroxidase [183]. The major issues with this approach are that it generates an analog output that cannot be standardized, is laboratory-based, and is heavily influenced by the presence of leukocytes. Nevertheless, this methodology has been used for diagnostic purposes in situations where appropriate reference values have been established for the local population [160]. However, the complexities created by leukocyte contamination are challenging and must be addressed when standardizing reference values for each scenario in the andrology laboratory setting.

### 7.2. Flow Cytometry

The confounding influence of leukocyte contamination has been resolved using flow cytometry. Using this procedure, gates can be set to accurately separate spermatozoa and leukocytes. When used with redox-sensitive dyes such as dihydroethidium (DHE) or DCFH-DA (2′,7′-dichlorodihydrofluorescein diacetate), the oxido-reductive status of the spermatozoa can be accurately determined [184]. Furthermore, specific probes, such as MitoSox Red, can be used to focus the analysis on mitochondrial ROS generation [185]. With the caveat that these probes more accurately measure oxidizing activity than specific ROS, the use of flow cytometry does appear to provide clinically useful information. Thus, in comparative studies, flow cytometry using MitoSox Red was the most efficacious probe for detecting the elevated ROS generation responsible for inducing DNA damage, and for detecting the oxidative stress exhibited by defective spermatozoa recovered from the low-density region of discontinuous density gradients [186]. This methodology was also effective in detecting the increase in ROS production observed following prolonged incubation in vitro [187]. It is clearly an effective probe for laboratory-based studies. However, the cost of providing and maintaining the infrastructure required to run such flow cytometry-based assays has hindered its widespread adoption as a routine diagnostic procedure.

The same could be said for flow cytometry assays of sperm DNA oxidation using 8-oxo-2′-deoxyguanosine as the target. This DNA base adduct is a recognized biomarker of oxidative stress that correlates well with semen quality parameters, leukocytospermia, and known drivers of oxidative stress, such as BMI and smoking [163,188]. However, the assay has not been widely used clinically, notwithstanding its evident value for laboratory-based research.

### 7.3. Oxido-Reductive Potential

To provide a routine point-of-care assay that might find application in infertility clinics, attention has instead focused on measuring seminal oxidation-reduction potential using the MiOXSYS system (Aytu BioScience, Englewood, NJ, USA) [189]. This technique measures the static ability of a given fluid to absorb or donate electrons; the major output is an sORP (static Oxidation-Reduction Potential) reading will be generated with elements of semen quality that are also correlated with count. If the analysis is confined to the sORP reading alone, then any correlation with semen quality is apparently lost [190,191]. An alternative electrochemical approach based on linear voltammetry has recently been described and provides a rapid assessment of the total antioxidant capacity of human semen, correlating well with sperm count. It will be interesting to see how this device performs in future clinical trials involving infertile patients [192].

### 7.4. Total Antioxidant Capacity

The fundamental notion that oxidative stress might be assessed not by measuring ROS (which are ephemeral and unstable) but by measuring the antioxidant status of human semen is supported by a large number of studies [192,193,194] that have demonstrated a significant inverse relationship between semen quality and Total Antioxidant Capacity (TAC). The colorimetric TAC assay is stable, inexpensive, and highly correlated with semen quality. Moreover, the assay has recently been simplified to generate a point-of-care diagnostic test suitable for clinical application [15]. Again, it will be interesting to see whether such technical developments result in a diagnostic assay that can be used to screen male infertility patients to determine who might benefit from antioxidant therapy and, just as importantly, when such therapy should cease once redox balance has been restored.

## 8. Conclusions

Human spermatozoa are highly differentiated cells that depend heavily on redox chemistry for their fundamental architecture and functional competence. The cross-linking of sperm chromatin during epididymal maturation, the capacitation of these cells in preparation for fertilization, the onset of hyperactivated movement, sperm–egg recognition, acrosomal exocytosis, and sperm–oocyte fusion have all been shown to involve redox-regulated pathways in one form or another. The fundamental ability of ROS to oxidize thiols underpins their capacity to crosslink protamines during sperm chromatin stabilization, to convert zymogens into active enzymes, to inactivate phosphatases, and to activate sAC, all of which are fundamental in this regard. However, these cells live their lives on a knife-edge. A wide variety of environmental, lifestyle, and clinical factors can induce excess ROS generation by sperm mitochondria and/or by enzymes such as NOX5 or NOS. When this occurs, the intrinsic vulnerability of these cells to oxidative stress becomes evident, and their structural and functional integrity is rapidly compromised. The creation of oxidative stress in this manner is thought to contribute to around one-third of all cases of male infertility [138]. While antioxidant therapy might be a rational approach for such patients, we are still some way from achieving this objective. There has been very little systematic work to determine the optimal composition of such a supplement, and we still lack a robust, simple, point-of-care diagnostic test to identify appropriate patients for treatment. Hopefully, ongoing research in this field will provide clinicians with the tools they need to detect and remedy oxidative stress in the male germ line. Since the impact of oxidative stress in the male germ line can have transgenerational effects by inducing DNA damage and increasing mutational load, such developments will not only improve the reproductive health of male patients but also ensure the health and well-being of future generations [195].

## Data Availability

No new data were created or analyzed in this study. Data sharing is not applicable to this article.
